# A218 INFLAMMATORY PHENOTYPE AS A PREDICTOR OF TREATMENT RESPONSE IN ULCERATIVE COLITIS

**DOI:** 10.1093/jcag/gwae059.218

**Published:** 2025-02-10

**Authors:** A AlDarwish, A Wilson

**Affiliations:** Gastroenterology, Western University, London, ON, Canada; Gastroenterology, Western University, London, ON, Canada

## Abstract

**Background:**

A variety of immune pathways contribute to the propagation of inflammation in ulcerative colitis (UC). Both, T_h_1- and T_h_2-associated cytokines and cells, have been implicated in the pathogenesis of the disease. Eosinophils, which are elicited by T_h_2 immune response, are increasingly recognized to have a pivotal role in the pathogenesis of UC, however, the exact link between eosinophils and UC disease outcomes and treatment responses over time is still incompletely defined.

**Aims:**

We aim to evaluate the association between pre-treatment peripheral eosinophil counts and treatment response in patients with ulcerative colitis.

**Methods:**

In this retrospective cohort study, data from 150 treatment naïve adult patients who were diagnosed with ulcerative colitis between 2005 and 2020 were reviewed. White blood count differential was collected at diagnosis and following exposure to one or more of the following treatments: corticosteroids, TNFa antagonists, or vedolizumab. Patients were stratified into two cohorts for analysis: “high-eosinophil” cohort (>=0.2x10^9^cells/L) and “low-eosinophil” cohort (<0.2x10^9^cells/L). The partial and endoscopic Mayo scores were evaluated at baseline and following treatment. Patients’ data were collected until their last gastroenterology follow-up or until surgical intervention. Demogoraphic data were summarized using descriptive statistics. Fisher’s Exact test was used to evaluate the association between eosinophil count and drug response as well as other outcomes.

**Results:**

A total of 150 patients were included. 92 patients (63.3%) were included in the high-eosinophil cohort, while 58 patients (38.6%) were included in the low-eosinophil cohort. The median duration of follow-up was 78 months (IQR42.5) and 74 months (IQR45) in the high-eosinophil group and low-eosinophil group, respectively. Between the high-eosinophil group and low eosinophil group, there was no statistically significant difference in terms of steroid response (71.3%, 78.6% respectively, p=0.330), achieving steroid-free remission with TNFa antagonist (76%, 72.2% respectively, p=0.662), achieving steroid-free remission with vedolizumab (54.1%, 65.2% respectively, p=0.394), or disease refractoriness requiring more than 2 drug changes (15.4%, 8.8% respectively, p = 0.106).

**Conclusions:**

This study did not show a correlation between baseline eosinophil count and response to therapy - corticosteroids, TNFa antagonist, and vedolizumab. Larger prospective studies are warranted to assess disease outcomes and treatment response to eosinophil-targeted therapy.

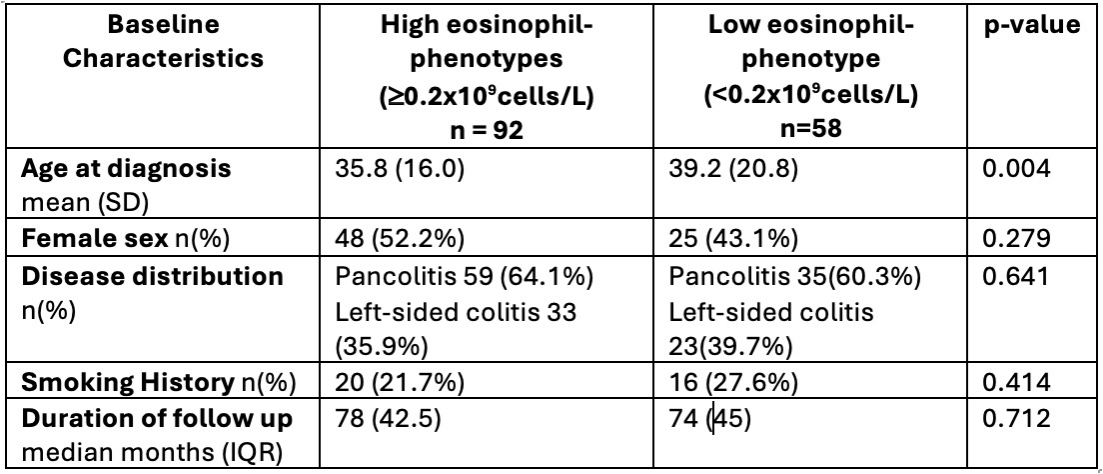

**Funding Agencies:**

None

